# Evaluation of different pretreatments on microbial transformation of saponins in *Dioscorea zingiberensis* for diosgenin production

**DOI:** 10.1080/13102818.2014.943019

**Published:** 2014-10-17

**Authors:** Tianxiang Zheng, Lidan Yu, Yuling Zhu, Bin Zhao

**Affiliations:** ^a^ College of Life Science, Shaoxing University, Shaoxing, China; ^b^ The State Key Laboratory of Hollow Fibre Membrane Materials and Processes, Tianjin Polytechnic University, Tianjin, China

**Keywords:** saponins, diosgenin, microbial transformation, pretreatment

## Abstract

In order to evaluate the effects of different pretreatments on microbial transformation of saponins in *Dioscorea zingiberensis* (DZW), various methods have been systematically studied on a large scale. Five pretreatments, including physical separation, catalytic solvent extraction, ultrasonic fermentation, complex enzymatic hydrolyzation and enzymatic saccharification, were performed on DZW. Compared with other methods, complex enzymatic hydrolyzation significantly improved the efficiency of microbial transformation. Due to the pretreatment, a diosgenin yield of 92.6%, and diosgenin accumulation of 27.3 mg/g DZW were achieved. The high efficiency of this method was attributed to the separation of 84.3% starch and 76.5% fibre from DZW in the form of a sugar. Analysis of saponins in this microbial transformation process showed that the residual rates of the intermediate products were much lower than those obtained from other pretreatments. The results demonstrate that complex enzymatic hydrolyzation is a practical and effective pretreatment method for production of diosgenin from DZW in a microbial transformation way.

## Introduction

Diosgenin, an important steroidal precursor used in the pharmaceutical industry, is widely used in synthesis of sex hormones, oral contraceptives and other steroid drugs.[1–3] It usually exists in plant tubers in forms of saponins attaching sugar chains to aglycone with C–O glycosidic bonds at C-3 and C-26.[4] Production of diosgenin from saponins mainly depends on hydrolyzation of the sugar chains at the two positions. Nowadays, microbial transformation is usually applied for this purpose because of its high specificity, mild reaction conditions and low cost.[5–8] However, the low diosgenin productivity restricts the application of microbial transformation.

*Dioscorea zingiberensis* C. H. Wright (DZW) is the main raw material for industrial diosgenin preparation in China because of the high saponins concentration in its tubers.[9] Starch (30%–40%, w/w), fibre (40%–50%) and saponins (2%–4%) make up about 95% of the entire composition of DZW.[10] Saponins in plant cells are wrapped in starch or fibre, which reduce the access of enzymes produced by microorganisms.[11,12] This is one factor that leads to the low efficiency of microbial transformation. Consequently, prior to microbial transformation, separation of starch and fibre from DZW is essential in order to improve the diosgenin productivity.[11–13] Until now, most researches in this field focused on recovery of starch and fibre from DZW,[14–16] and few studies concerned about releasing saponins from the wrap of starch or fibre. To loosen the cell walls of DZW, a variety of physical, chemical and biological pretreatments can be applied. Establishment of the best method to maximize the diosgenin production via biotransformation was worth studying.[17]

The objective of this work was to investigate the effects of different pretreatment methods (physical separation, catalytic solvent extraction, ultrasonic fermentation, complex enzymatic hydrolyzation and enzymatic saccharification) on microbial transformation. The composition of each pretreated DZW method was carefully analysed with starch, reducing sugar, fibre (hemicellulose, cellulose and lignin) and saponins (total saponins, diosgenin-diglucoside, disogenin-rhamnoside-glucoside, diosgenin-glucoside and diosgenin) contents. The pretreated DZW tubes were then subjected to microbial transformation with *Trichoderma reesei* (*T. reesei*) for 168 h on a large scale. The saponins produced in the microbial fermentation process were determined, and the best result was applied for diosgenin production.

## Materials and methods

### Materials

DZW tubers were from ShiYan City, Hubei Province in China. α-Amylase (2000 IU/g), saccharifying enzyme (10,000 IU/g) and cellulase (10,000 IU/g) were purchased from Shandong Longda Bio-product Co., Ltd., Shangdong, China. Alcohol dehydrogenase (ADH) yeast was from Angel Yeast Company, Yichang, Hubei, China. The standard of diosgenin-3-O-[β-D-glucopyranosyl(1→4)]-α-L-rhamnopynosyl (diosgenin-glucoside-rhamnoside) was supplied by Nantong Kanmaike Co., Ltd (Jiangsu, China). The standards of diosgenin-3-O-[β-D-glucopyranosyl(1→4)]-β-D-glucopyranside (diosgenin-diglucoside) and trillin (diosgenin-glucoside) were from Zhongkangweiye Co., Ltd. (Shaihai, China). All other chemicals that were used were analytical regent grade unless stated different.

### Microorganism

The fungal strain *T. reesei* (ACCC 30597) was conserved in the Agricultural Culture collection of China (Beijing, China) and acclimated with 50% (w/v) of multi-saponins extracted from DZW. The strain was stored on potato dextrose agar slant at 4 °C and sub-cultured every two weeks.

To prepare the inoculum, fungal spores in a seven-day-old agar were suspended in 5 mL of 0.01% (w/v) Tween 80 solution and added to 100 mL of medium (30 g sucrose, 3 g NaNO_3_, 1 g K_2_HPO_4_, 0.5 g MgSO_4_, 0.01 g FeSO_4_, 0.1 g Tween 80 and 1000 mL water). Fungal cells were incubated at 30 °C on a temperature controlled shaking incubator at 150 rpm for 6–7 days until mycelia mats were observed.

### Pretreatment methods

**Figure 1.**
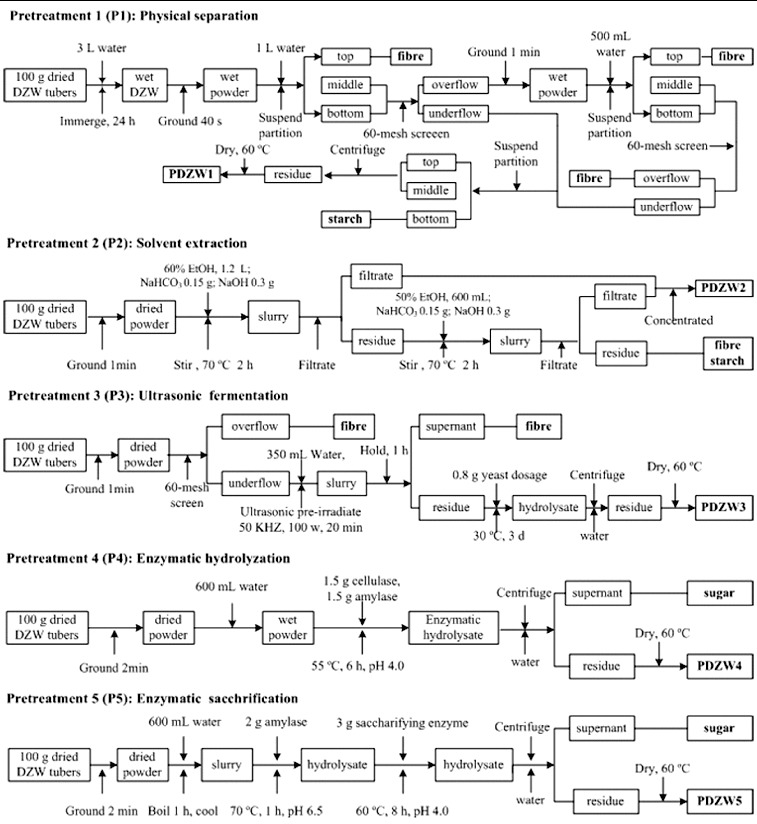
Flow charts of pretreatment methods used in the experiments.

DZW tubers were cleaned and dried at 60 °C. Before microbial transformation, the raw material was subjected to various pretreatment methods to recover starch and fibre, including physical separation (P1), catalytic solvent extraction (P2), ultrasonic fermentation (P3), complex enzymatic hydrolyzation (P4) and enzymatic saccharification (P5). Practical procedure of each pretreatment method was described in details in Figure 1. The experiments were conducted in a 10 L reaction vessel containing 6 L medium. A control experiment that did not include any pretreatment was also carried out. Before microbial transformation, the DZW and obtained pretreated DZW (PDZW) were dried at 60 °C and grounded to pass through a 60-mesh screen. The contents of starch, reducing sugar, fibre [12] and saponins in DZW and PDZW were measured.

### Microbial transformation

Microbial transformation was carried out using DZW or PDZW as substrate on a large scale. The experiments were conducted in a 5 L stirred bioreactor containing 3 L medium (2.67% peptone, 0.27% K_2_HPO_4_, 0.73% Tween 80, 10% substrate, w/v, pH 5.8).[18] The bioreactor was autoclaved at 121 °C for 15 min, inoculated with 300 mL of sub-cultured fungal spore suspension containing 10^7^ spores/mL and incubated at 30 °C, at an aeration of 0.80 vvm and agitation rate of 300 rpm for 168 h as we previously reported.[7] Every 12 h, 30 mL of the sample was taken out. Saponins (diosgenin-diglucoside, diosgenin-glucoside-rhamnoside, diosgenin-glucoside and diosgenin) in the microbial transformation broth were determined.

### Analysis

#### Total saponins

Total saponins in DZW and PDZW were determined in the form of diosgenin. An amount of 10 g substrate was hydrolysed with 30 mL 1 mol/L H_2_SO_4_ at 121 °C for 3 h, then filtered and washed with water until neutral pH was reached. The residue was dried at 60 °C and the content of diosgenin in it was determined with HPLC.[11]

#### Saponins

The fermentation sample (15 mL) was centrifuged and dried at 60 °C. 10 mL of n-BuOH was added to each sample and ultrasonicated for 30 min. After filtration, the n-BuOH extract was concentrated to dryness with rotary evaporator. The dried extract was dissolved in 2 mL of methanol.

Diosgenin-glucoside-rhamnoside, diosgenin-diglucoside, diosgenin-glucoside and diosgenin in the extract were analysed with an Agilent 1100 HPLC coupled with Agilent MSD Ion Trap. The sample was separated on a Zorbax Eclipse XDB-C18 Liquid chromatography (LC) column (Agilent 2.1 × 150 mm, particle size 5 μm, pore size 80 Å, monomeric double-endcapped) at 40 °C, with a flow rate of 1 mL/min. The mobile phase was consisted of water (A) and CH_3_CN (B). The gradient programme was 50%–75% (B, v/v) for the first 15 min, 75%–92% (B, v/v) for 15–17 min and 92% (B, v/v) for the last 16 min. The mass scan was in the range of m/z 400–1000 for negative ion mode.

The residual rate of diosgenin-glucoside-rhamnoside (C1), diosgenin-diglucoside (C2), diosgenin-glucoside (C3) and the diosgenin (C4) yield were calculated with the following equations:(1) 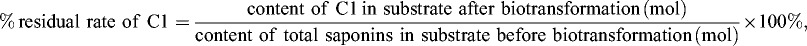




(2) 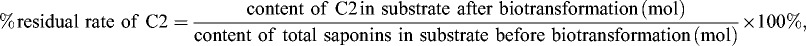

(3) 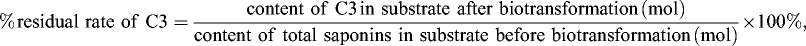

(4) 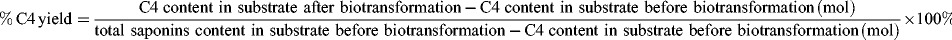



## Results and discussion

### Effects of pretreatments on composition of DZW

The effects of different pretreatment methods on the properties of the DZW are shown in Table 1. The untreated DZW tubers in this study contained about 36.8% starch and 49.8% fibre (hemicellulose, cellulose and lignin). Removal of starch and fibre from the plant tubers is one important part of the pretreatment. It is related to starch and fibre being available carbon sources in the fermentation industry but wasted when DZW is directly used for diosgenin preparation.[16,19] Another reason for the importance of pretreatment is that recovery of starch and fibre from DZW can release saponins from the network of cell walls, which makes them more accessible to enzymes. The five pretreatments could remove starch from DZW, and the most effective methods were P3 and P5, which recovered about 95% starch from DZW tubers in the form of sugars. With respect to fibre, P2 was the most effective method for removing hemicellulose, cellulose and lignin from DZW, while P4 could also effectively recover hemicellulose and cellulose in the form of sugars from the raw material.

**Table 1.** Composition of different pretreated DZW (in 100 g dry matter)*.

	Untreated	P1	P2	P3	P4	P5
Weight (g)	100^a^	43.9 ± 3.15^b^	20.3 ± 1.78^d^	33.2 ± 2.61^c^	25.1 ± 3.45^d^	34.4 ± 3.91^c^
Starch (g)	36.8 ± 1.59^a^	13.5 ± 1.20^b^	10.4 ± 2.00^c^	1.42 ± 0.17^e^	5.78 ± 0.61^d^	1.78 ± 0.15^e^
Reducing sugar (g)	5.36 ± 0.32^a^	4.68 ± 0.50^ab^	4.49 ± 0.52^b^	0.74 ± 0.22^d^	2.66 ± 0.54^c^	1.04 ± 0.12^d^
Hemicellulose (g)	40.0 ± 1.83^a^	10.2 ± 0.96^c^	2.48 ± 0.41^e^	18.4 ± 1.19^b^	5.56 ± 0.14^d^	17.4 ± 0.98^b^
Cellulose (g)	2.40 ± 0.29^a^	2.11 ± 0.08^a^	0^c^	1.03 ± 0.18^b^	1.11 ± 0.16^b^	1.33 ± 0.24^b^
Lignin (g)	7.44 ± 0.52^a^	6.06 ± 0.25^b^	0^d^	5.25 ± 0.45^c^	5.06 ± 0.11^c^	7.00 ± 0.12^a^
Total saponins (g)	2.78 ± 0.18^b^	2.13 ± 0.24^c^	2.65 ± 0.19^b^	2.96 ± 0.19^a^	2.94 ± 0.15^a^	2.97 ± 0.17^a^

*Experiments were done in triplicate. Data in the table are mean ± standard deviation.

^a–e^Indication letters. Values in the same raw not sharing the same letters are significantly different (one-way ANOVA with SNK method, *p* < 0.05).

Prevention of saponins losses in PDZW is another important part of the pretreatment. About 2.78% of total saponins were detected in the raw material. P1 resulted in the lowest saponins concentration of 2.13%, because some saponins were wrapped in recovered starch and lost in the grinding and screening process. The contents of saponins in PDZW3, PDZW4 and PDZW5 increased by 10% compared with DZW, respectively. The higher saponin concentrations in PDZW3, PDZW4 and PDZW5 can be explained by the enzymes’ coactions, which liberate saponins from starch and fibre.

Although the concentrations of saponins in PDZW3, PDZW4 and PDZW5 were at the same levels, the saponins compositions in the three substrates were different (Table 2). For example, PDZW4 contained about 0.85% diosgenin-diglucoside, 0.23% diosgenin-glucoside-rhamnoside, 0.061% diosgenin-glucoside and 0.072% diosgenin while about 0.46% diosgenin-diglucoside, 0.078% diosgenin-glucoside-rhamnoside, 0.048% diosgenin-glucoside and 0.039% diosgenin were detected in PDZW5. This is because cellulase in P4 could not only hydrolyse hemicellulose, cellulose in DZW, but also could break the links between diosgenin and sugar chains in saponins.

**Table 2.** Steroid saponins in different pretreated DZW (in 100 g dry matter).

	Diosgenin-diglucoside*	Diosgenin-rhamnoside-glucoside	Diosgenin-glucoside	diosgenin
Untreated	0.37 ± 0.04^d^	0^c^	0^c^	0^d^
P1	0.36 ± 0.02^d^	0^c^	0^c^	0^d^
P2	0.47 ± 0.05^c^	0^c^	0^c^	0^d^
P3	0.66 ± 0.05^b^	0.074 ± 0.01^b^	0.051 ± 0.004^b^	0.022 ± 0.003^c^
P4	0.85 ± 0.03^a^	0.23 ± 0.003^a^	0.061 ± 0.007^a^	0.072 ± 0.008^a^
P5	0.46 ± 0.03^c^	0.078 ± 0.009^b^	0.048 ± 0.008^b^	0.039 ± 0.004^b^

*Unit: g.

^a^ ^–d^Indication letters. Values in the same column not sharing the same letters are significantly different (one-way ANOVA with SNK method, *p* < 0.05).

### Effects of pretreatments on biotransformation of saponins

Until now, many saponins in DZW have been isolated and identified, e.g. zingibernsis new saponin, deltonin, diosgenin-triglucoside, dioscin, progracillin, diosgenin-diglucoside and so on.[5] Qualitative and quantitative analyses of the saponins involved in the microbial transformation were difficult and time-consuming. Our prior studies demonstrated that sugar chains in the C-3 hydroxyl group in saponins were released from the aglycone stepwisely by *T. reesei*.[11] LC-MS (Liquid chromatography-Mass spectrometry) analysis showed that diosgenin-diglucoside, diosgenin-rhamnoside-glucoside, diosgenin-glucoside and disogenin were the main intermediate products in the fermentation broth during the microbial transformation. Therefore, these four compounds were the main saponins also analysed in this study by us.

**Figure 2.**
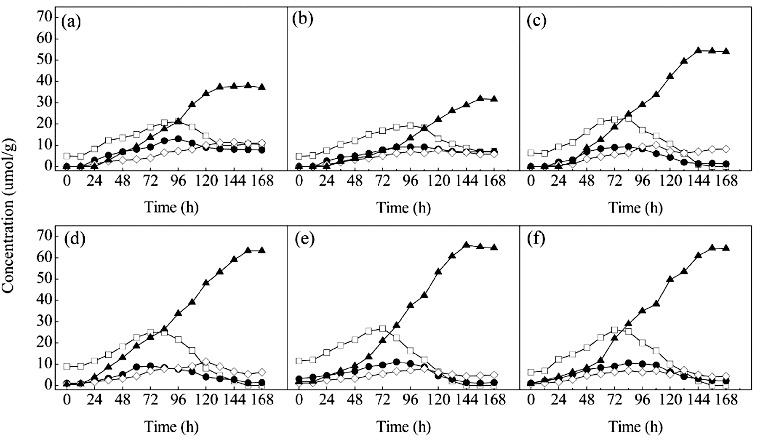
Time course of the diosgenin-diglucoside (□), diosgenin-rhamnoside-glucoside (•), diosgenin-glucoside (⋄) and diosgenin (▴) in bioreactor cultivation of DZW (a), PDZW1 (b), PDZW2 (c), PDZW3 (d), PDZW4 (e) and PDZW5 (f) by *T. reesei*. Data were expressed as mean value. The standard deviations were less than 10%.

When *T. reesei* was grown on DZW (Figure 2(a)), the concentrations of diosgenin-diglucoside and diosgenin-glucoside-rhamnoside which were yielded from saponins with longer sugar chains and then transformed to less polar ones with shorter sugar chains slowly increased in the first 96 h. The content of diosgenin-glucoside was increased from 12 to 96 h, and the concentration of diosgenin still remained low during this period. Thus, the first 96 h appeared to be the production period for intermediate products. From 96 to 156 h, the concentrations of diosgenin-diglucoside and diosgenin-glucoside-rhamnoside slightly decreased. Diosgenin continuously increased to a maximum of 37.9 μmol/g. This period could be described as the production time for diosgenin. At 156 h, the main saponins in the fermentation broth were diosgenin. Diosgenin-diglucoside, diosgenin-glucoside-rhamnoside and diosgenin-glucoside were the three main by-products in this process.

The changes of the concentrations of the saponins produced from PDZW were similar to that from DZW. However, the contents of diosgenin accumulated from PDZW3, PDZW 4 and PDZW5 were 63.2, 65.9, 64.4 μmol/g DZW, significantly higher than that from DZW (Figure 2(b)–(f)).

**Figure 3.**
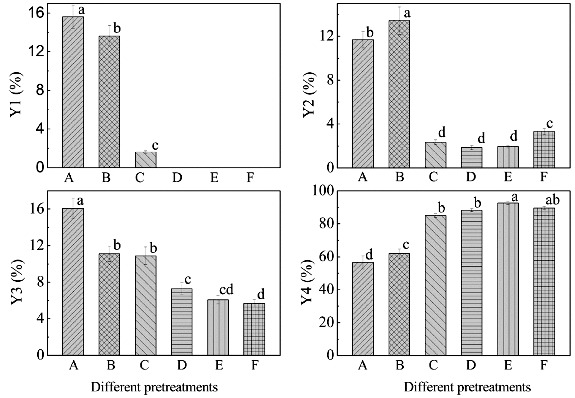
Residual rate of diosgenin-diglucoside (Y1), residual rate of diosgenin-glucoside-rhamnoside (Y2), residual rate of diosgenin-glucoside (Y3) and diosgenin yield (Y4) of the microbial transformed DZW and PDZW. A: DZW; B: PDZW1; C: PDZW2; D: PDZW3; E: PDZW4; F: PDZW5. Values not sharing the same letters are significantly different (one-way ANOVA with SNK method, *p* < 0.05).

As shown in Figure 3, pretreatment could increase the diosgenin yield of biotransformation in varying degrees. Of the five pretreatment methods, complex enzymatic hydrolyzation resulted in the highest diosgenin yield of 92.6%, in contrast to the lowest diosgenin yield of 56.5% that was obtained under control (no treatment) conditions. The diosgenin yield was 62.0%, 85.0%, 88.3% and 89.6% for P1, P2, P3 and P5, respectively.

Low residual rate of diosgenin-diglucoside (0%), diosgenin-glucoside-rhamnoside (1.96%) and diosgenin-glucoside (6.08%) in the fermentation broth of PDZW4 at 156 h were accounted for the highest diosgenin yield (Figure 3). In this microbial transformation phase, during the first 96 h, the fungal biomass slowly increased, lack of carbon source in PDZW4 induced the fungi to produce more enzymes, and simultaneously, the diosgenin sharply increased. Production of diosgenin from DZW was related to some extent to the glucosidase activity. Previous study demonstrated that α-rhamnase and β-glucosidase not only exhibited excellent performances on the hydrolysis of the terminal α-L-rhamnose or β-D-glucose in sugar chains, but also cleaved the glycosidic linkage between aglycone and β-D-glucose. The diosgenin yield was determined by the activities and stereo-selectivities of the enzymes in the system.[20,21] This was confirmed by the results achieved with other substrates. When *T. reesei* was incubated with PDZW3, the residual rates of diosgenin-diglucoside, diosgenin-glucoside-rhamnoside and diosgenin-glucoside were 0%, 1.85% and 7.31%, respectively, and the diosgenin yield was 88.3%. When PDZW1 was transformed with *T. reesei*, the residual rates of diosgenin-diglucoside, diosgenin-glucoside-rhamnoside and diosgenin-glucoside in the fermentation broth were 13.6%, 13.4% and 11.1%, respectively, and the diosgenin yield was 62.0%.

## Conclusions

The effect of various pretreatments on production of diosgenin from DZW with microbial transformation method was systematically investigated. The results demonstrated that biological pretreatments provided better results in terms of diosgenin production than physical or chemical methods. Among the biological methods in this research, the complex enzymatic hydrolyzation was determined as the best pretreatment for diosgenin production with *T. reesei*. This method resulted in 84.3% of starch and 76.5% of fibre that were recovered from DZW in the form of sugars. An optimal diosgenin production with a diosgenin yield of 92.6%, and diosgenin accumulation of 27.3 mg/g DZW were obtained, which was 39.0% higher than that achieved from direct microbial transformation of raw materials. This study will be of great significance in production of diosgenin from plant tuber in an environmental-friendly and technological-efficient way in the industry.
